# Randomized, Controlled Intervention Trial of Male Circumcision for Reduction of HIV Infection Risk: The ANRS 1265 Trial

**DOI:** 10.1371/journal.pmed.0020298

**Published:** 2005-10-25

**Authors:** Bertran Auvert, Dirk Taljaard, Emmanuel Lagarde, Joëlle Sobngwi-Tambekou, Rémi Sitta, Adrian Puren

**Affiliations:** **1**Hôpital Ambroise-Paré, Assitance Publique—Hôpitaux de Paris, Boulogne, France,; **2**INSERM U 687, Saint-Maurice, France,; **3**University Versailles Saint-Quentin, Versailles, France,; **4**IFR 69, Villejuif, France,; **5**Progressus, Johannesburg, South Africa,; **6**National Institute for Communicable Disease, Johannesburg, South Africa; San Francisco General Hospital, San Francisco, CaliforniaUnited States of America.

## Abstract

**Background:**

Observational studies suggest that male circumcision may provide protection against HIV-1 infection. A randomized, controlled intervention trial was conducted in a general population of South Africa to test this hypothesis.

**Methods and Findings:**

A total of 3,274 uncircumcised men, aged 18–24 y, were randomized to a control or an intervention group with follow-up visits at months 3, 12, and 21. Male circumcision was offered to the intervention group immediately after randomization and to the control group at the end of the follow-up. The grouped censored data were analyzed in intention-to-treat, univariate and multivariate, analyses, using piecewise exponential, proportional hazards models. Rate ratios (RR) of HIV incidence were determined with 95% CI. Protection against HIV infection was calculated as 1 − RR. The trial was stopped at the interim analysis, and the mean (interquartile range) follow-up was 18.1 mo (13.0–21.0) when the data were analyzed. There were 20 HIV infections (incidence rate = 0.85 per 100 person-years) in the intervention group and 49 (2.1 per 100 person-years) in the control group, corresponding to an RR of 0.40 (95% CI: 0.24%–0.68%; *p* < 0.001). This RR corresponds to a protection of 60% (95% CI: 32%–76%). When controlling for behavioural factors, including sexual behaviour that increased slightly in the intervention group, condom use, and health-seeking behaviour, the protection was of 61% (95% CI: 34%–77%).

**Conclusion:**

Male circumcision provides a degree of protection against acquiring HIV infection, equivalent to what a vaccine of high efficacy would have achieved. Male circumcision may provide an important way of reducing the spread of HIV infection in sub-Saharan Africa. (Preliminary and partial results were presented at the International AIDS Society 2005 Conference, on 26 July 2005, in Rio de Janeiro, Brazil.)

## Abstract 2

The first trial of male circumcision for reducing the risk of HIV finds significantly lower new cases in the treatment group.

## Introduction

Male circumcision (MC) is associated with various cultural factors, including religious sacrifice, rites of passage into adulthood, and the promotion of hygiene. The earliest documentary evidence for circumcision is from Egypt. Tomb artwork from the Sixth Dynasty (2345–2181 B.C.) shows circumcised men, and one relief from this period shows the rite being performed on a standing adult male. Genesis (17:11) places the origin of the rite among the Jews in the age of Abraham, who lived around 2000 B.C.

Presently, MC practices in Africa are varied. Whereas men in Muslim countries are circumcised, as in North Africa or a large part of West Africa, in other societies the prevalence of MC depends on other cultural factors, such as changes that occurred under colonization. In countries such as Cameroon and the Democratic Republic of Congo, which are predominately non-Muslim, most men are circumcised [1–3]. In Kenya, where only a minority of men are Muslims, men in all tribes except the Luo practice MC [4].

The first paper suggesting a protective effect of MC against HIV infection was published in 1986 [5]. Since then, many observational studies have been published, some of which have observed that most men living in East and southern Africa, the regions with the highest prevalence of HIV, are not circumcised [1–3]. A majority of these observational studies are cross-sectional, and a minority are prospective [6–11]. A systematic review and meta-analysis found that in sub-Saharan Africa MC is associated with a significantly reduced risk of HIV infection among men, with an adjusted relative risk of 0.42 (95% CI: 0.34%–0.54%) [12].

All of these studies were based on observational data, and, in the absence of experimental studies, a causal relationship between MC and protection against HIV infection could not be determined [13]. Direct experimental evidence is needed to establish this relationship and, should a protective effect of MC be proven, to convince public health policy makers of the role of MC in reducing the spread of HIV [7,13,14].

The primary objective of this study was to determine the impact of MC on the acquisition of HIV by young men through a randomized, controlled, blindly evaluated intervention trial. The secondary objective was to assess the role of behavioural factors known to be associated with HIV serostatus in explaining the possible impact. This study was conducted in the Gauteng province of South Africa, where HIV prevalence among pregnant women was estimated to be 29.6% in 2003 [15]. According to an earlier study in the research site area, 59% (95% CI: 55%–63%) of uncircumcised men said that they would be circumcised if it reduced their chance of acquiring HIV and STDs [16].

## Methods

### General Presentation

A randomized, controlled, blindly evaluated intervention trial was carried out in Orange Farm and surrounding areas, a semi-urban region close to the city of Johannesburg. The recruitment of participants took place in the general population from July 2002 to February 2004. Information about the trial was disseminated in the community through meetings during the recruitment period. Precise oral and written information was delivered at the investigation centre to potential participants during a pre-screen visit. Participants were then informed that the impact of MC on the acquisition of sexually transmitted infections (STIs), including HIV, is not known. A minimum of 3 d after the pre-screen visit, potential participants were screened for eligibility. Potential participants with genital ulcerations were temporarily excluded until successful treatment. The inclusion and exclusion criteria are listed in Table 1. The participants received a total of 300 South African Rand as compensation (1 South African Rand ~ 0.12 Euro). The protocol, the consent form, and the participant information sheet are provided as Text S1–S3.

**Table 1:**
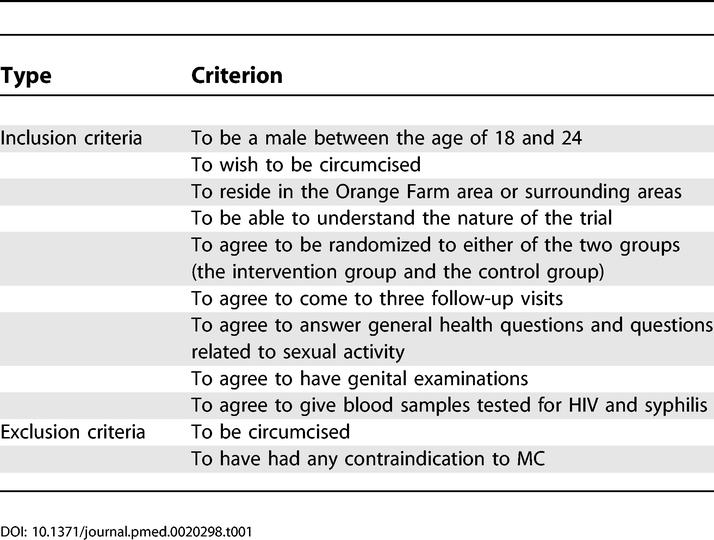
Inclusion and Exclusion Criteria

### Randomization

At the end of the screen visit, following screening and written consent, participants were divided into two groups, using sealed envelopes. Participants requested to participate actively in the random assignment. Consequently, each participant was invited by the manager of the centre to choose an envelope containing the group name from a basket of ten envelopes. After each randomization, a new envelope was added to the basket. This added envelope was taken sequentially from a set of envelopes pre-prepared in such a way that each set of envelopes contained five for the “Control” and five for the “Intervention” arm. Participants of the intervention group were offered to be circumcised within a week. Participants of the control group were asked to wait until the end of the trial before being offered to be circumcised.

### Follow-Up and Data Collection

After the screen visit, which took place at month 1 (M1), the three follow-up visits took place at the end of M3, M12, and M21. The M3 visit was designed to study the possible impact of surgery on HIV acquisition as a result of sexual activity during the healing phase following circumcision or contamination during surgery. These three follow-up visits defined three sequential periods, M1–M3, M4–M12, and M13–M21, with expected durations of 3, 9, and 9 mo, respectively. The duration of these periods was measured in days from the dates of the visits, the day after the end of a period being the beginning of the next period.

A participant lost to follow-up was defined as a participant who had not completed a planned visit in the 2 mo following the planned date of this visit and who did not complete any further visit. A missing visit was defined as a visit not completed prior to a completed visit.

At each of the four visits, each participant was invited to answer a face-to-face questionnaire, to provide a blood sample, and to have a genital examination and an individual counselling session. The questionnaire allowed for collection of data on background characteristics and reported sexual behaviour. The last section of the questionnaire allowed for the description of all sexual partnerships over the previous 3 mo for the M3 visit and over the previous 12 mo for all other visits. This section allowed each participant to describe the number of sexual contacts, the date of first and last sexual contact, the frequency of condom use (never, sometimes, always), and the type of partnership (spousal or non-spousal), a spousal partner being defined as a sexual partner with whom the respondent is married or living as married. Characteristics of sexual behaviour during the 9-mo periods M4–M12 and M13–M21 were determined from this section, using the dates of first and last sexual contact of each sexual partner. The genital examination was performed by a trained nurse who recorded the circumcision status and took a blood sample from each participant. Blood samples were tested for syphilis and HIV-1.

The counselling session (15–20 min) was delivered by a certified counsellor and focused on information about STIs in general and HIV in particular and on how to prevent the risk of infection. During this session, participants were encouraged to attend voluntary counselling and testing in a public clinic located 200 m away from the investigation centre or in a voluntary counselling and testing (VCT) centre funded by the project and located in the same building as the investigation centre. Condoms were provided in the waiting room of the investigation centre and were also provided by the counsellor. Participants who had symptoms of STIs, as assessed by the nurse during the genital examination, or who tested positive for syphilis were treated at the local clinic or by doctors working for the project. A specific programme for prevention of opportunistic infections and delivery of antiretroviral treatment, if required, was put in place at the VCT centre to assist participants who attended VCT and who tested positive for HIV. The arrangement will remain in place until the public sector programme becomes operational in the area.

The standard of care in South Africa at the beginning of the trial in July 2002 included VCT but not access to antiretroviral therapy. With the formal introduction of access to antiretroviral therapy in 2004, there were increased efforts to encourage participants to attend VCT and referrals to appropriate facilities were instituted. In this context, it was decided to include participants independent of VCT attendance. Consideration for making HIV testing compulsory for participation in the trial or recruiting only those who tested HIV-negative would certainly lead to stigmatization, and the investigators considered that the whole concept of VCT was that it should be voluntary. They considered it unethical to inform participants of their HIV status without their permission, even if they thought that participants should be aware of their HIV status. They also considered it unethical to deter from participating in the study potentially at-risk men who did not want to know their HIV status. Indeed, HIV-positive participants would benefit from the trial: (a) by receiving counselling at each visit, (b) by undergoing clinical examination and syphilis testing, and (c) by having a medicalized circumcision that could possibly protect them or their sexual partners against other STIs or even against re-infection by HIV.

### Male Circumcision

The median (interquartile range [IQR]) duration between randomization and MC was one day (0–2). The circumcisions were performed by three local general practitioners in their surgical offices. The general practitioners were experienced in MC practices. The cost of each circumcision was 300 South African Rand and was paid for by the project. The procedure was standardized and used the forceps-guided method, as is widely practiced in South Africa, and was reviewed by the Department of Urology, University of the Witwatersrand Medical School, South Africa.

### Quality of the Data, Blinding, Confidentiality, and Data Management

To ensure confidentiality, participants' files were kept in a locked room at the centre and each participant received a number that was used to identify all documents related to that person. To ensure blinding of study personnel, the randomization group information was not available to the personnel in charge of counselling or collecting information in the centre during the participants' visits.

Questionnaires were checked at the end of each interview. Participants failing to turn up for any follow-up visit were visited at home by trial staff, who encouraged them to come for the follow-up visits or ascertained the reasons for dropping out.

Laboratory results were stored in a database that was independent of the one used to store the information related to each participant. During the study, no HIV results were available to the investigation centre or to the investigators, apart from the statistician in charge of the interim analysis.

Laboratory results and data collected from questionnaires were entered twice in a database (Microsoft Access, Redmond, Washington, United States) by different people. The two entries were compared, and discrepancies were corrected. The data were then re-checked for inconsistencies using the source documents. After the data had been cleaned, they were imported into the statistical package SPSS for Windows version 8 (SPSS, Chicago, Illinois, United States) and R (version 2.0.1) for analysis [17].

### Laboratory Procedures

Following the interview, a trained nurse collected whole blood samples in the investigation centre. One EDTA blood tube of 10 ml of venous blood was taken and immediately centrifuged at 400 g for 10 min, and five aliquots of plasma were frozen at −20 °C. The samples were identified only by the participant number and transported each week to the laboratory, where they were stored at −70 °C and tested.

An ELISA screen (Genscreen HIV1/2 version 2, Bio-Rad, France) and two ELISA confirmatory tests (Wellcozyme HIV recombinant, Abbott Murex, Dartford, United Kingdom, and Vironostika HIV Uni-Formm II plus O, bioMérieux, Boxtel, Netherlands) were used to test plasma for HIV-1 infection. Samples that were positive on all three ELISAs were regarded as “positive” and all others as “negative” [18].

### Ethics

The research protocol was reviewed and approved by the University of Witwatersrand Human Research Ethics Committee (Medical) on 22 February 2002 (protocol study no. M020104). The trial was also approved by the Scientific Commission of the French National Agency for AIDS Research (ANRS; protocol study no. 1265; 2002, decision No. 50) and obtained authorization from the City of Johannesburg, Region 11, on 25 February 2002. A Data and Safety Monitoring Board was responsible for analyzing adverse events and for deciding on the results of the interim analysis.

### Adverse Events

Adverse events (AEs) were documented and analyzed for all participants, including those who were HIV-positive at randomization. These AEs related to surgery, and that occurred in the first month post-surgery, were reported by the practitioners using a specific form. In addition, at each visit to the centre the nurse completed a questionnaire after the genital examination to record adverse events. During home visits for missing participants, any deaths were recorded.

### Sample Size and Interim Analysis

The total sample size was initially calculated to be 2,580 HIV-negative participants in order to obtain a power of 80% to detect a 50% reduction in the proportion of HIV infection between the groups at a 5% significance level, assuming an HIV incidence of 2.2 per 100 person-years (py) in the control group. This number, calculated using Fisher's exact test, was increased to 3,035 to account for 15% of participants lost to follow-up. An interim analysis was planned for when all the M12 visits had been completed, and this was conducted blind with the database obtained on 29 November 2004. At the time of the interim analysis, the total follow-up included an estimated 63% of the total number of py that would have been collected at the end of the study, leading to a threshold value of 0.0095, as determined by the Lan-DeMets alpha-spending function method [19].

### Statistical Analysis

While participants with a HIV-positive test at M1 were followed in the same way as the other participants, they were excluded from the statistical analysis. HIV status was considered as censored data with time being continuous, observed in a grouped form (at the end of each period), with non-uniform duration of periods. These data were modelled using a piecewise exponential, proportional hazards model in which the baseline hazard is constant in each period. This theoretical model allows the precise duration between each visit and time-dependent covariate to be taken into account. It was implemented by running a Poisson log-linear model on a dataset composed of lines corresponding to the periods M1–M3, M4–M12, and M13–M21, in which the participant stayed HIV-negative or became HIV-positive [20–22]. Consequently, in this dataset, each individual was represented by a maximum of three lines. This type of model gives an incidence rate and incidence rate ratio (RR) of HIV infection among men of the intervention group in comparison with men of the control group. The protection against HIV infection was calculated as 1 − RR.

At the interim analysis, the RR was 0.37 in the intervention group, as compared with the control group, with a *p* value of 0.00073, below the threshold value. The Data and Safety Monitoring Board advised the investigators to interrupt the trial and offer circumcision to the control group, who were then asked to come to the investigation centre, where MC was advised and proposed. The database corresponding to planned visits up to 30 April 2005 was then analyzed, and the results are presented in this paper. Because the study was interrupted, some participants did not have a full follow-up on that date, and their visits that were not yet completed are described as “planned” in this article.

Adjusted rates and RRs were obtained by taking into account covariates that were calculated for each period when they were time-dependent. Three nested models were developed. The model-1 included the period number, which was included as categorical variables, with the logarithm of the duration of exposure in each period in days as an offset. In the model-2, the calendar period of recruiting and background characteristics of the participants were added. In the model-3, behavioural time-dependent covariates, characterizing the behaviour of participants during each period, were also added.

The background characteristics of the participants considered were age (less than or equal to 21 y, more than 21 y), religion (Catholic or Protestant, African traditional, other), ethnic group (Zulu, Sotho, other), and alcohol consumption in the past month. The five reported sexual behaviour covariates considered were, for each period of follow-up, being at-risk behaviour (defined as having at least one sexual contact unprotected by condom), having a spousal partner, the number of non-spousal sexual partners, the number of sexual contacts, having at least one relationship with only one sexual contact. In addition, health-seeking behaviour was characterized by at least one visit to a clinic for a genital problem during the 12-mo period prior to a visit to the centre.

Additional analyses were also performed. (a) The impact of the intervention was assessed among those having completed their M21 visit. (b) The impact of the intervention on participants who were 1 mo or more late to at least one follow-up visit or missed one follow-up visit was compared with the impact of the intervention on other participants by testing the corresponding interaction term between this factor and the randomization group. (c) To analyze the impact of the 6-wk period of abstinence, the analysis was repeated with the duration of the period M1–M3 reduced by 42 d in the intervention group. Forty-two days was the median (IQR = 28–56) interval between MC and first sexual contact reported by sexually experienced participants of the intervention group. (d) The effects of MC across the ethnic groups were studied by assessing this impact among the two major ethnic groups of this study (Zulus and Sothos) and by testing the corresponding interaction term. (e) Finally, while all analyses were performed in intention-to-treat, a per-protocol analysis was performed using the circumcision status observed at each visit.

Six comparisons of the behavioural factors for each of the periods M4–M12 and M13–M21 were performed. Independence of behavioural categorical data between the randomization groups was tested using Fisher's exact test, and the Kruskal-Wallis test was used for quantitative behavioural variables. Assuming that these comparisons were independent, and to keep the overall risk of type I error equal to 0.05, the level of significance was set as 1.00 − 0.95 ^1/6^ = 0.0085.

## Results

**Figure 1:**
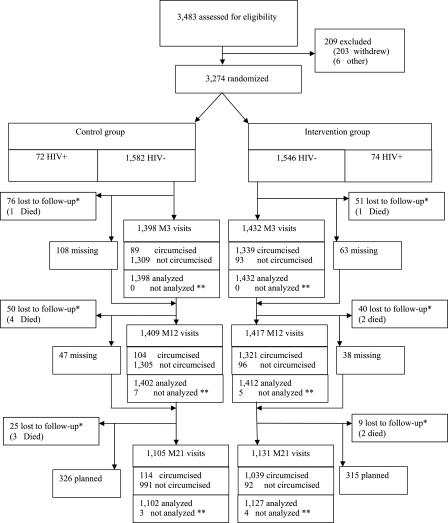
Trial Profile This figure describes the state of the trial corresponding to planned visits up to 30 April 2005. HIV-positive and HIV-negative participants were randomized. All were followed, but only participants HIV-negative at randomization were analyzed and are represented in the three follow-up visits of the figure. After randomization, the participants could attend the 3-mo visit, miss it, or be excluded from follow-up (death or loss to follow-up). The non-excluded participants who attended the 3-mo visit could then attend the 12-mo visit, miss it, or be excluded (death or loss to follow-up). The non-excluded participants of the 12-mo visit could then attend the 21-mo visit, be excluded (death or loss to follow-up) or were planning to attend the 21-mo visit but had not yet done so, because of the interruption of the trial. *, did not come for the scheduled visit (refused, withdrew, moved away or died); **, no blood sample

Table 2 gives the baseline characteristics for the HIV-negative participants. The median age (IQR) was 21.0 y (19.6–22.5). Most of the participants had completed the primary level of education. Very few were married or living as married, and about half were at-risk behaviour. Figure 1 shows the trial flowchart. A total of 3,274 men participated in the trial. There were 146 (prevalence 4.5%) HIV-positive participants at randomization. The difference in size between the intervention and control group was 34 (1,620 versus 1,654).

**Table 2:**
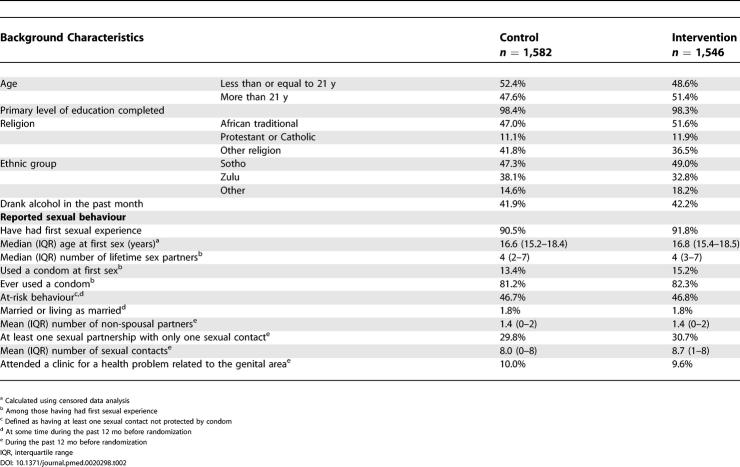
Baseline Characteristics of HIV-Negative Men Enrolled in the Trial

Among the 3,128 HIV-negative participants at randomization, the visits at M3, M12, and M21 took place at (median; IQR) 3.0 (3.0–3.2), 12.0 (11.9–12.1), and 20.9 mo (20.9–21.2) after randomization, respectively. The mean (IQR) follow-up was 18.1 mo (13.0–21.0).

The fraction of participants lost to follow-up was 8.0 % (251/3128), with 6.5% (100/1546) in the intervention group and 9.5% (151/1582) in the control groups (*p* = 0.0016, Fisher's exact test). Among the participants lost to follow-up at the visit M12 or M21, none (0/124) were HIV-positive at their previous completed visit.

During the study, 20 and 49 participants acquired HIV infection in the intervention and control groups, respectively, corresponding to incidence rates (95% CI) of 0.85 per 100 py (0.55–1.32) and 2.1 per 100 py (1.6–2.8) in the intervention and control groups, respectively. Using model-1, the RR of HIV infection for the intervention group in comparison with the control group was 0.40 (0.24–0.68), *p* = 0.00059 (Table 3). This RR corresponds to a protection of 60% (32–76) against HIV infection. This result is equivalent to saying that during the period M1–M21 the intervention prevented six out of ten potential infections.

**Table 3:**
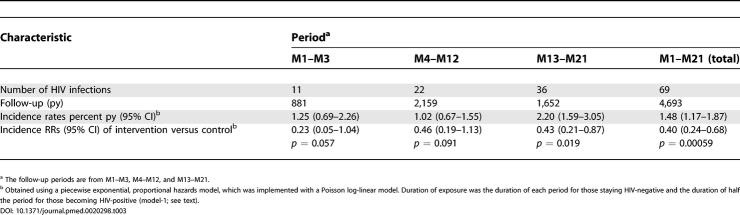
Characteristics of the Follow-Up Period

When considering only those participants who completed their M21 visit, the RR was 0.38 (0.22–0.67), *p* < 0.001. In comparison with the others, those who were 1 mo or more late to at least one follow-up visit or missed one follow-up visit (1178/3128; 37.7%) had the same risk of HIV infection (RR = 1.06; 0.65–1.73; *p* = 0.82) and were not differently protected by MC (*p* = 0.69). When reducing the M1–M3 period by 42 d in the intervention group, the RR was RR = 0.43 (0.26–0.73), *p* = 0.0016, a value close to the RR obtained in the intention-to-treat analysis. This indicates that the 6-wk period of abstinence plays a minor role in explaining the effect of the intervention during the period M1–M21. Among the two major ethnic groups of the participants, Zulus (*n* = 1,109) and Sothos (*n* = 1,506), the RR was 0.60 (0.25–1.41), *p* = 0.24, and 0.42 (0.20–0.88), *p* = 0.022, respectively. These two RRs were not significantly different (*p* = 0.55). The per-protocol analysis gave RR = 0.24 (0.14–0.44), *p* < 0.001, a value lower to the RR obtained in the intention-to-treat analysis. The difference of the results given by the two analyses is at least partly explained by the cross-overs. In the intervention group, 6.5% (93/1432) were not circumcised at M3, and in the control group, 10.3% (114/1105) were circumcised at M21 (Figure 1).

For the periods M1–M3, M4–M12, and M13–M21, the number of HIV infections was two, seven, and 11 in the intervention group and nine, 15, and 25 in the control group. The RR for each of these periods is given in Table 3. In the period M1–M3, there was an RR of 0.23 close to the significance level, which was slightly higher when taking into account the 42 d of abstinence (RR = 0.37; 0.08–1.72; *p* = 0.21).

Using model-2, an RR was found similar to that obtained with model-1: 0.38 (0.23–0.65), *p* < 0.001. This result is attributable to the randomization process, which distributed the characteristics equally between the intervention and control groups.

Of the five reported sexual behavioural factors, all were higher in the intervention group than in the control group during the period M4–M12, and four out of five were higher during the period M13–M21. Only the mean number of sexual contacts showed statistically significant differences during the period M4–M12 (5.9 versus 5.0, *p* < 0.001) and during the period M13–M21 (7.5 versus 6.4, *p* = 0.0015). The proportion of participants attending a clinic for a genital problem in the 12 mo prior to M12 was lower in the intervention group than in the control group (4.7% versus 7.2%, *p* = 0.0067).

Using model-3, the RR, adjusted on behavioural characteristics, reported by participants during the follow-up is similar to the RR obtained with model-1 (Table 4). This last result indicates that the protective effect of the intervention is not attributable to the change of reported behaviour associated with the intervention and shows that adjustment for potential confounders has little effect on the association of MC and HIV incidence.

**Table 4:**
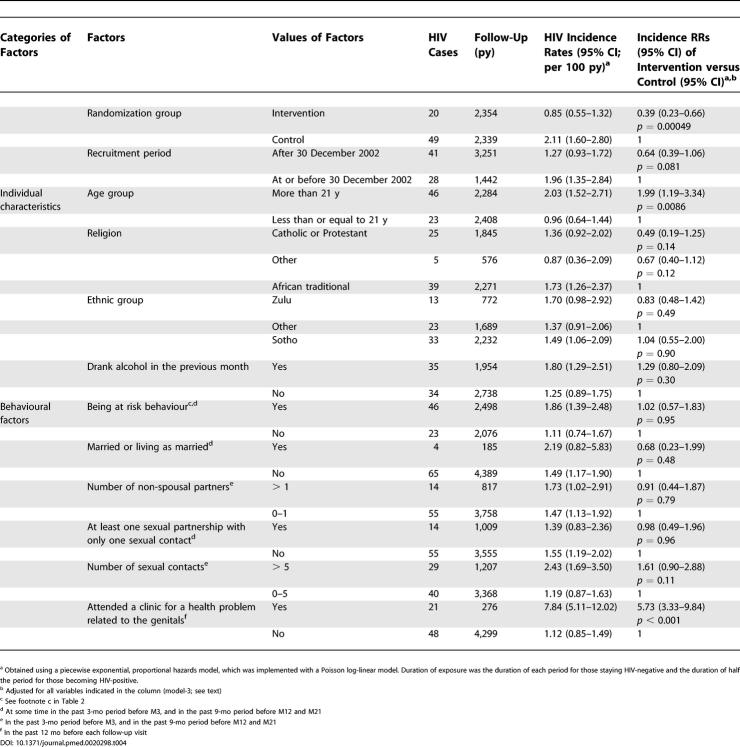
Multivariate RRs of HIV Incidence

**Figure 2:**
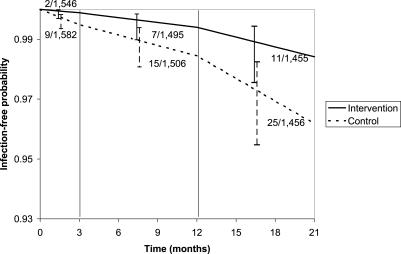
Infection-Free Probability As a Function of Time and of Randomization This figure represents the infection-free probability using a piecewise exponential distribution with boundaries at M3, M12, and M21 obtained with a Poisson log-linear model (see text). Each segment of exponential has been fitted to the data in each period for each randomization group. The 95% confidence intervals have been represented in the middle of each period. x/y is the number of HIV infections observed in each period (x) and the number of persons at the beginning of the period (y).

Figure 2 shows the fitted infection-free probability as a function of time and of randomization. Table 5 describes the 60 (3.8%) AEs that were reported during surgery or in the first month following surgery among 1,568 MCs performed in the intervention group, HIV-positive at randomization included. The proportion of AE was higher among those who were HIV-positive at randomization, and the difference is close to significance (*p* = 0.056, Fisher's exact test). At M3, 98.5% of those who were circumcised (HIV-positive at randomization included), were “very satisfied” with the result of the circumcision. Adverse events recorded at the end of the follow-up (M21) are described in Table 6.

**Table 5:**
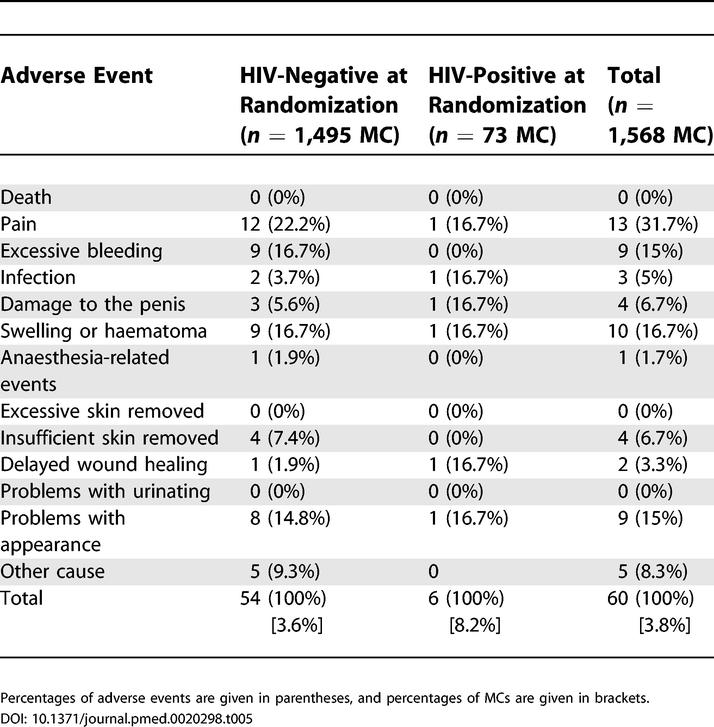
Adverse Events during Surgery or in the First Month following Surgery among Those Having Been Randomized in the Intervention Group, as a Function of HIV Status at Randomization

**Table 6:**
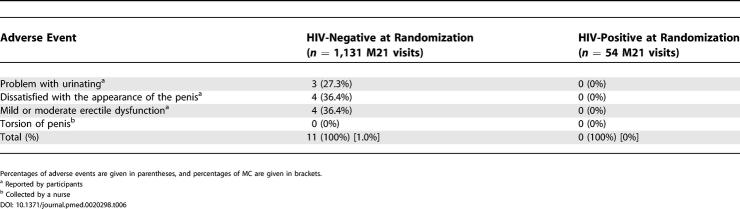
Adverse Events at the End of the Follow-Up (M21) among Those Having Been Randomized in the Intervention Group, As a Function of HIV Status at Randomization

Home visits for late participants revealed 16 deaths among participants (HIV-positive at randomization included), of whom six had been circumcised, but examination of death certificates, reports from doctors who carried out the MC, interviews with relatives, and timing of these deaths revealed no deaths related to MC. The mortality rate from the South African Census 2001 data [23] in the age groups 15–19 and 20–24 for the black population of the Gauteng province was 2.4 and 3.9 per 1,000 per year. These figures lead to an estimate of 3.5 per 1,000 per year at the mean age (21.0 y) of our participants. In turn, this value leads to an estimated number of deaths of 15.7 using the mean follow-up, which is close to the number of deaths observed in our trial.

## Discussion

This study provides the first experimental evidence of the efficacy of MC in protecting men against HIV infection. It was conducted in a general population, and it is the first randomized control trial testing the impact on health of MC. The demonstration in this study of a causal association between HIV infection and MC is consistent with protection suggested by meta-analyses of observational studies [12] but with a higher protective effect. This difference can be explained, at least partly, by the effect of bias and confounding factors associated with cross-sectional studies. High values ranging from 0.12 to 0.29 of protective effect of MC have been reported in prospective studies conducted in high-risk groups [6,8–11]. Our study is also the first experimental study demonstrating that surgery can be used to prevent an infectious disease. In addition, this finding is an a posteriori proof of the use of MC to improve hygiene in the common meaning of not being infected.

This study has some limitations. It was conducted in one area in sub-Saharan Africa and, therefore, may not be generalizable to other places. Nevertheless, because of the similar route of transmission of HIV in sub-Saharan Africa and because observational studies from various areas of sub-Saharan Africa have shown an association between HIV status and MC [12], the result of this trial is applicable to all of sub-Saharan Africa with some degree of confidence.

Even though some participants were lost during the follow-up, and the loss to follow-up rate was greater than the event rate, the impact of missing participants on the overall results of this study is likely to be small not only because the loss to follow-up was small for a cohort study conducted in a general population, but also because those who were late for at least one follow-up visit were protected by MC just as the other participants. The reason for this loss to follow-up was a result of participants moving from the area or being unreachable, and not a result of HIV infection.

Because the Data and Safety Monitoring Board recommended to stop the trial after the intermediate analysis, it was not possible to follow all the participants as initially planned, and, as a consequence, only those participants recruited at the beginning had a full follow-up. This potential bias was taken into account by adjusting the analysis for the recruitment period; such an adjustment cannot fully account for the confounding effect associated with partial follow-up. When restricting the analysis to those participants who had a full follow-up, the intervention had an effect that was similar in size and significance, suggesting that this potential bias had a negligible impact.

A specific survey was implemented after the end of the recruiting period in order to assess the satisfaction of the results of the randomization. Of the participants, 65.3% said they were happy. However, the results also showed that a limited number of participants (7.5%), strongly unhappy with their group of randomization, were allocated and recorded in the other group. They were analyzed in their randomization group in the intention-to-treat analysis. The findings were confirmed by the person in charge of randomization. This factor contributed to increase the cross-over, which remained low, and to dilute the measure of the effect of the intervention, which remained high.

Another limitation concerns the timescale of this study. Participants were followed up for a short period of time, and, therefore, this study did not explore the long-term protective effect of MC.

The protective effect of MC on HIV infection was unchanged when controlling for sexual behaviour, including condom use, which was taken into account when defining those at-risk behaviour, the period of abstinence in the intervention group following MC, and heath-seeking behaviour, which was considered because treatment of STIs can have an effect on HIV acquisition [24]. This shows that these factors play a minor role in explaining the protective effect of MC on HIV infection. The reasons for this protective effect of MC on HIV acquisition have to be found elsewhere, and several direct or indirect factors may explain this [25]. Direct factors may be keratinization of the glans when not protected by the foreskin, short drying after sexual contact, reducing the life expectancy of HIV on the penis after sexual contact with an HIV-positive partner, reduction of the total surface of the skin of the penis, and reduction of target cells, which are numerous on the foreskin [26]. Indirect factors may be a reduction in acquisition of other STIs, which in turn will reduce the acquisition of HIV. Our study does not allow for identification of the mechanism(s) of the protective effect of MC on HIV acquisition.

The first and obvious consequence of this study is that MC should be recognized as an important means to reduce the risk of males becoming infected by HIV. As shown by our study, MC is useful and feasible even among sexually experienced men living in an area with high HIV prevalence. Indeed, in our study the intervention delivered by local general practitioners resulted in a limited and reasonable number of adverse events and did not lead to an increase in deaths. In addition to the protective role in men, MC will indirectly protect women and, therefore, children from HIV infection because if men are less susceptible to HIV acquisition, women will be less exposed. Moreover, MC may also be protective against male-to-female HIV transmission, but this will require further investigation [7]. The role that women can play in promoting MC is potentially important. If women are aware of the protective effect of MC, this awareness could, in turn, have an impact on the prevalence of MC by encouraging males to become circumcised.

It was found that the protective effect of MC is high. MC provides a degree of protection against acquiring HIV infection equivalent to what a vaccine of high efficacy would have achieved. Consequently, the authors think that MC should be regarded as an important public health intervention for preventing the spread of HIV. MC could be incorporated rapidly into the national plans of countries where most males are not circumcised and where the spread of HIV is mainly heterosexual. This is even more important at a time when no vaccine or microbicides are currently available and when delivering antiretroviral treatments under WHO guidelines will have only a small impact on the spread of HIV [27]. In addition, MC is an inexpensive means of prevention, performed only once, and men can be circumcised over a wide age range, from childhood to adulthood.

The potential impact of prevention programmes based on MC is difficult to assess at population level and requires modelling. From the results of this study and of the meta-analysis quoted above, it can be predicted that widespread MC could lead to a strong reduction of the spread of HIV. The availability of a simple and ancient practice with a high potential effect on the spread of HIV is remarkable and should encourage decision makers to take MC into consideration as policy. Because most of southern and East Africa is concerned, the number of HIV infections that could be avoided by the widespread implementation of MC is high.

There are potential risks in promoting MC as way of reducing the risk of HIV infection. MC can be performed under poor hygienic conditions, leading to not only infection, bleeding, and permanent injury, but also HIV infection from non-sterilized instruments, and possible death if appropriate treatment of sequelae is not provided. In the healing period, sexually active men are likely to be at a higher risk of HIV infection, and this risk should not be underestimated. MC does not provide full protection and, if perceived as full protection, could lead to reduction of protection of men who, for example, decrease their condom use or otherwise engage in riskier behaviour. It was found that the intervention group had significantly more sexual contacts. While the protective effect of circumcision remained despite this increased risk, this should be a concern when considering implementation of circumcision as a means of preventing HIV infection. Finally, there is the danger of confusing MC with female circumcision, and that promotion of MC could be used by defenders of female circumcision to defend this practice.

Acceptability studies of the use of MC as a prevention measure against the spread of HIV have been conducted in South Africa [16,28], Kenya [29,30], Zimbabwe [31], and Botswana [32]. These studies, in which most of the uncircumcised African men expressed interest in becoming circumcised if performed safely and affordably, highlighted the potential of MC as a population-level intervention to reduce HIV spread. MC is a not a universal cultural practice, and cultural practices can be barriers in policy considerations. However, there are examples showing that the prevalence of MC can be changed. For example, in South Korea 50 years ago, almost no men were circumcised; today some 85% of Korean men 16–29 y old are circumcised [33].

The experimental demonstration of the protective effect of MC on the acquisition of HIV emphasizes the role of MC in explaining the heterogeneity of HIV prevalence in sub-Saharan Africa. From a multi-site study conducted in four African countries, MC, together with sexual behaviour, has been posited as an important factor in the heterogeneity of HIV prevalence in sub-Saharan Africa [34]. This role is confirmed and reinforced by the findings of the present study.

## Supporting Information

Text S1 Effect of Medicalized Male Circumcision on the Incidence of HIV, Herpes Simplex Virus 2, and Genital Ulcerations (204 KB PDF).

Text S2 Consent Form (29 KB PDF).

Text S3 Participant Information Sheet (45 KB PDF).

**Patient Summary**

**Background**

HIV/AIDS is one of the greatest threats to health worldwide. More than 3 million people died of AIDS last year, and about 5 million others became infected with HIV, bringing the total number of people living with the infection to nearly 40 million. The situation is particularly severe in Africa, which has 10% of the world's population but two-thirds of the world's people with HIV. In many African tribal groups, men are circumcised, usually in late childhood or early adolescence, and this is an important part of their cultural identity. In other African ethnic groups, men are not circumcised. By the late 1980s, researchers noticed that HIV infection rates were lower in those tribes where men were circumcised. But it was not clear whether it was circumcision itself or some other difference in behaviour between the circumcised and uncircumcised groups that gave some protection to the circumcised men against getting HIV.

**What Did The Researchers Do?**

The researchers wanted to find out whether circumcising men could reduce their chance of becoming infected by HIV. They offered young, sexually active, heterosexual, uncircumcised men in Johannesburg, South Africa, the chance to have the operation. They explained that half of those who came forward would be circumcised right away (the “treatment group”) and the other half would be circumcised 21 months later (the “control group”). Some 3,000 men joined the study. The group that each man was put into was decided at random. The plan was that all the men would visit the research clinic four times during this 21-month period, and that they would be tested for HIV each time. However, after 14 months, the number of new infections in the control group (49) was so much greater than the number in the treatment group (20) that it was considered unethical to continue the study. (The men in the control group were told they could be circumcised without any further delay.)

**What Do These Findings Mean?**

Infections were 60% fewer in the treatment group, which seems to indicate that circumcised men are much less likely to become infected with HIV when having sex with infected women. In communities where HIV is common, circumcision may prove to be a valuable tool for reducing men's risk of getting infected. However, as with most studies, criticisms could be made of some aspects of the methods used, and more research is needed before we can be sure. We must also remember that circumcised men can still become infected, even though the risk might be lower. They should still take other steps to prevent themselves from getting HIV.

**Where Can I Get More Information Online?**

The United Nations health agencies, including the WHO and UNAIDS, issued a statement when this research was first presented at a meeting in Brazil in July 2005:

http://www.who.int/mediacentre/news/releases/2005/pr32/en/

UNAIDS (http://www.unaids.org) has information about the state of the HIV/AIDS epidemic and prevention strategies. It produces an annual report and has documents on a wide range of topics. The Q&A documents are particularly useful:

http://www.unaids.org/EN/resources/questions_answers.asp#II

Many organizations provide information on AIDS prevention—for example, the Terrence Higgins Trust:

http://www.tht.org.uk

AEGIS is the world's largest searchable database on HIV and AIDS:

http://www.aegis.com

## Abbreviations

AE: adverse event
IQR: interquartile range
M[number]: month [number]
MC: male circumcision
py: person-year
RR: rate ratio
STI: sexually transmitted infection
VCT: voluntary counselling and testing
