# Correction: Randomized, Controlled Intervention Trial of Male Circumcision for Reduction of HIV Infection Risk: The ANRS 1265 Trial

**DOI:** 10.1371/journal.pmed.0030226

**Published:** 2006-05-30

**Authors:** Bertran Auvert, Dirk Taljaard, Emmanuel Lagarde, Joëlle Sobngwi-Tambekou, Rémi Sitta, Adrian Puren

In *PLoS Medicine* volume 2, issue 11: DOI: 10.1371/journal.pmed.0020298

Table 4: Under “Religion,” the values for “HIV cases,” “Follow-up,” and “HIV incidence rates” for the categories “Catholic or Protestant” and “Others” were reversed in error. The correct values are as follows.

Catholic or Protestant: HIV cases, 5; Follow-up (py), 576; HIV Incidence Rates (95% CI per 100 py), 0.87 (0.33–2.09).

Others: HIV cases, 25; Follow-up (py), 1,845; HIV Incidence Rates (95% CI per 100 py), 1.36 (0.92–2.02).

