# Supplementary material for: Randomized, Controlled Intervention Trial of Male Circumcision for Reduction of HIV Infection Risk: The ANRS 1265 Trial
Source: PLoS Med. 2005 Oct 25;2(11):e298. doi: 10.1371/journal.pmed.0020298 (PMC1262556; doi:10.1371/journal.pmed.0020298)
Supplement: Supplementary file 1 [file pmed.0020298.st001.pdf]

Effects of medicalized male circumcision on the incidence of HIV, herpes simplex virus-2 (HSV-2) infections and genital ulceration

A intervention study in South Africa

30-April-2002

Contents

1 Introduction
- HIV and male circumcision in sub-Saharan Africa
- The Role of male circumcision in the fight against HIV in Africa
- Male circumcision in sub-Saharan Africa
- Arguments invoked by adversaries of male circumcision
- Experimental demonstration is required
- International Context
- Male circumcision and HIV in South Africa, more particularly in Carletonville

2 Objectives and design
- Main hypothesis
- Main objective
- Secondary objectives
- Study design
- Duration of study
- Inclusion criteria
- Exclusion criteria
- Medicalized male circumcision
- Main and secondary criteria of assessment
- Side effects
- Randomization
- Questionnaires
- Laboratory tests and clinical examination
- Counseling
- Participant follow-up
- Analysis stages
- Biases reduction
- Data confidentiality
- Statistical Analyzes
- Population characteristics
- Number of subjects
- Feasibility and selection methods
- Management
- Budget
- Ethical Considerations
  - *Justification of study*
  - *Selection of participants independently of their HIV or HSV-2 status*
  - *HIV Tests*
  - *Before study*
  - *During study*
  - *At the end of the study*

3 Management
- Membership
- Scientific Council
- Independent Monitoring Committee

Plan of the study

References

Author: Pr. Bertran Auvert INSERM U88 14, rue du Val d'Osne
94415 Saint-Maurice Cedex France
Tel.: 01 45 18 38 71 bertran.auvert@paris-ouest.univ-paris5.fr

Summary

Infection rate of HIV is high among young men. The objectives of this study are to measure the effect of an intervention of safe male circumcision on the incidence rate of infection by sexually transmitted infections, including HIV. A group of 3520 voluntary men, aged from 18 to 24 years, will be followed for 21 months. Safe male circumcision will be offered to half of the participants at the beginning of the study, and will be offered at the end of the study to the second half. The result of this intervention will be essential to discuss the interest of safe male circumcision in the fight against HIV in South Africa.

1 Introduction

HIV and circumcision in sub-Saharan Africa

The HIV epidemic reaches a catastrophic width in sub-Saharan Africa. This epidemic is marked by a mainly heterosexual transmission and a geographical heterogeneity. The protective role of male circumcision was highlighted by ecological studies [1-3], cross-sectional studies [4-11] and longitudinal studies [12-16].
The protective role of male circumcision on HIV transmission from women to men is now largely recognized and accepted by the international scientific community. A recent meta-analyze showed indeed that circumcised men are about twice less infected than uncircumcised ones [17]. Moreover, the analysis of a multi-centre cross-sectional study conducted in Benin, Cameroon, Kenya and Zambia showed that male circumcision is a key factor to explain the differences in HIV prevalence in sub-Saharan Africa [18]. Finally, two studies analyzed the role of age at male circumcision on the protective effect of circumcision against HIV infection [19, 20]. The results are contradictory. The first study showed a protective effect of male circumcision even when performed after 13 years and the second one, however of limited power, did not demonstrate it.

At last, some studies showed a protective effect of male circumcision against ulcerative sexually transmitted diseases (STD) [21-23] that are risk factors of HIV infection. [10, 24-27].

Mechanisms of the male circumcision protective effect are not clearly highlighted. Several factors appear to play a role [28, 29]: 1) circumcision would protect from acquiring ulcerative STDs which are known to support HIV transmission 2) the foreskin would be a favorable ground for bacteria, 2) the foreskin is subject to lesions (abrasions, erosions) during sexual intercourse, 3) the foreskin is an area of strong density of Langerhans cells, which are HIV portal of entry.

The role of circumcision in the fight against HIV in Africa

Means for the fight against the spread of HIV in Africa are limited. The use of condoms appears to be stagnant: 20% of protected sex, what is probably insufficient for an effective impact [30]. Sexual behavior change such as reducing the number of sexual partners, which is part of all HIV fight national programs seems to have been successful in Uganda only [31]. Many hopes rest currently on antiretroviral drugs diffusion. However, availability of antiretroviral drugs is at an experimental stage and comes across financial and management problems. National programs of fight against STD do not seem to meet the expected success. Last of all, circumcision is not currently considered as an effective prevention method by people in charge of national programs, as well as by international agencies. In this context, the

experimental demonstration of a protective effect of male circumcision against HIV would be very significant in the fight against this disease in Africa.

Circumcision in sub-Saharan Africa

The circumcision distribution in sub-Saharan Africa is heterogeneous [2, 32]. In some countries of Africa, nearly all men are circumcised (example: Cameroon, Benin, Democratic Republic of Congo) while in other countries the proportion is varying. Traditionally, the practice of circumcision is related to the religious and ethnical membership. Thus, all Moslems are circumcised and the proportion of circumcised men generally varies according to their ethnical membership. But in Africa, it is noteworthy that in many countries, circumcision is a national practice mostly exceeding the ethnical or religious context. Thus, in Cameroon and Benin, more than 99% of men are circumcised whereas Moslems only account for about 10% of the population [11]. The current practices relating to circumcision might be closely related to the colonizers attitudes towards them. In Asia, there is one example of a foreign presence resulting in some modifications in the circumcision practices. Thus, in South Korea where people generally undergo circumcision at an age of 15-20 years, the circumcision prevalence grew among young men from 10% to 85% under the USA influence. It is thus clear that circumcision can play a significant role of public health intervention to fight against HIV.

Arguments raised by male circumcision adversaries

Adversaries of circumcision have many arguments. They are proposing that 1) It is impossible to integrate circumcision in national programs because it is a religious or ethnical practice, that cannot thus be modified; 2) There is a risk of mixing circumcision up with excision and promoting the first could give arguments to the excision supporters; 3) There is a risk of conviction that circumcision is an absolute protection which would thus result in increasing sexual activity, reducing the use of condoms and so favoring HIV spread; 4) Circumcision is usually performed in such hygiene conditions to increase this operation increases the risk for complications and thus ruin expected benefits; 5) Circumcision is a mutilating practice that cannot thus be performed by physicians except in some medical indications such as phimosis or repeated STDs; 6) Circumcision is a mutilating practice which cannot thus be performed before adulthood in order to obtain free and informed consent from the patient; 7) Circumcised men have a better genital hygiene than uncircumcised ones, which would explain the protective effect of circumcision; thus genital hygiene requires more attention than circumcision. The first argument does not resist when one examines the circumcision practices in Africa. Neither does the seventh one; In fact, in the South African area where the trial could be undertaken, there is a high level of masculine genital hygiene [33]. Public Health is concerned with arguments n° 2, 3 and 4 (feasibility, impact and cost/effectiveness analyses) which exceed the limits of this protocol; in any case they could only be discussed if the result of this study showed a protective effect of circumcision. This protocol holds account of arguments n° 3, 4 and 6. The protocol indeed will include only men aged more than 18 years, circumcisions will be performed by trained physicians in order to reduce operative risks to a minimum; moreover the possible impact of circumcision on sexual behavior – including the use of condoms - will be studied in detail.

Need for an experimental demonstration

All surveys concerning the effect of circumcision on HIV infection and other STDs are observational surveys. No experimental study has ever been led. In order to contribute to the Public health debate concerning the place of circumcision in prevention strategies, it is crucial to specify the protective effect (if there is) of circumcision when performed in the conditions

which will be met in this intervention study: that is an operation carried out by physicians (so we call it “medicalized circumcision” in this paper) before the age when men are strongly exposed to HIV infection. One could argue against the need for this trial, that it is not essential since the literature results are enough to prove the circumcision effectiveness. We do not agree with this argument. In our opinion indeed, there is on the contrary a risk of seeing HIV infection increase during the practice of medicalized circumcision (by ways of increasing sexual activity while men are convinced to be totally protected by circumcision); moreover from a scientific point of view, cross-sectional descriptive studies are lower in the hierarchy of evidence than randomized controlled trials.

International context

This project of randomized controlled trial takes place in a context of international competition and collaboration. Currently, an analogous project of controlled and randomized trial, supported by National Institutes of Health (NIH), will start at the end of 2001 in Kisumu (Kenya). The Director of this trial is Prof Robert Bailey (USA). The Kenya trial and the project proposed in this document were drawn up in cooperation. The team in charge of this project and Prof Bailey’s team have exchanged their protocols, and adapted them in order to enable a comparative study of the results. A British team (Prof Richard Beam) is looking for a site to carry out an analogous trial. Moreover, another team in North America (RH Gray) is considering setting up a similar trial in Rakai (Uganda). This protocol was designed to be realized in a duration well-suited with the international context.

Male circumcision and HIV in South Africa, and to Carletonville in particular

The HIV epidemic has reached a very high level in South Africa, since the estimated prevalence was 24.5% in October 2000 among pregnant women [34]. The South African epidemic is recent since this prevalence was lower than 1% in 1990. Yet, no published survey showed a protective effect of circumcision in this country.

The practice of male circumcision which is heterogeneous in South Africa, is well documented for the site of Carletonville only, which will be one of this protocol sites. In this city, the team in charge of the project has just carried out a cross-sectional population-based epidemiologic study (n=606) [35] combined with a qualitative survey; they showed that 1) the prevalence of male circumcision is about 20% 2) circumcision is performed in an ethnical context and the rate of circumcised men varies with the tribe (mainly: Sotho: 37%; Xhosa: 62%; Tswana: 15%; Zulu: 13%) 3) about 42% of circumcisions are carried out by physicians; 4) attending the initiation schools does not mean that circumcision won’t be performed by physicians; 5) the median age at male circumcision is 18 years; and 6) Most of the uncircumcised men in this inquiry (59%) declared they would agree to be circumcised if that could reduce their risk of HIV or other STD infections.

2 Objectives and design

Main hypothesis
The main hypothesis, which will be tested, is that male circumcision performed by physicians results in a 50% reduction of the incidence of HIV infection relative to the HIV incidence in uncircumcised men.

Main objective
The main objective is to measure the protective effect of the medicalized male circumcision on HIV infection in men aged 18-24 years in South Africa.

Secondary objectives
The secondary objectives are to measure the protective effect on infections by the genital herpes agent (Herpes simplex virus type 2 - HSV-2) and also on the incidence of genital ulcer disease.

Study Design
This study is a randomized controlled intervention trial. This multi-centre study will take place in 3 centers located around Johannesburg, in the areas of Orange Farm, Sebokeng and Evaton. The intervention group patients (circumcised at the beginning of the trial) and the control group (uncircumcised men) will be followed during 21 months (from M.0 to M. 21). Randomization and medicalized circumcision will be performed at M.0 in the intervention group and might be optional in the control group at end of study. The medicalized circumcision effectiveness will be evaluated on and after M.3 (3 months after medicalized circumcision). Incidences (of HIV, HSV-2 infections and genital ulcer disease) will be compared from M.3 to M.21 between the intervention group and the control group. An intermediate analysis will take place at M. 12.

Duration of study
Participants will be involved for 21 months (from M.0 to M.21). The period of inclusion is foreseen to last 9 months. The total duration of study should be thus 30 months.

Inclusion Criteria
- Uncircumcised men aged 18-24 years (the “no circumcision” criterion will be checked according to the method suggested by E.L. Wynder [36])
- in good general condition with normal physical and genital examinations
- Consenting to participate in the trial and specifically:
    - Consenting to randomization of the medicalized circumcision schedule (performed at the beginning of study for the treated group, optional at the end of study for the control group)
    - Consenting to avoid sexual contacts (except with condom protection) during the 6 weeks following the medicalized circumcision
    - Consenting to blood tests at M.0, M.3, M.12 and M.21, and HIV and HSV-2 tests.
    - Consenting to report any genital ulcer appearing between M.3 and M.21

Non-inclusion Criteria
- Circumcised men
- Men with AIDS
- Men with contraindication for circumcision

- Men thinking of moving away from the trial sites within the 21 months following inclusion
- Men with clinical STDs (those men could be included after treatment)

Medicalized male circumcision
Trained general practitioners will perform circumcision through a standard protocol developed by the Department of Urology of the University of Witwatersrand. This protocol in particular will include a medical post-circumcision exam.

Main and secondary criteria for assessment

The main criterion for assessment will be the number of HIV infections among the M.0 HIV-negative participants within the M.0-M.21 period.

Secondary criteria are 1) the number of HSV-2 infections among the M.0 HSV-2 negative participants within the M.0-M.21 period, 2) the number of genital ulcerations detected within the M.0-M.21 period, 3) sexual behavior and condom use during the M.0-M.21 period (number of new partners, number of short-term partners, number of sex acts, proportion of condom protected sex acts within non-spousal relationships).

Those criteria will be compared between the 2 groups of the study.

Adverse effects
All medicalized circumcision adverse effects should be formerly listed (mild, moderate, severe, life-threatening complications); when occurring they would be duly recorded and reported at once to the Independent Monitoring Committee (IMC). They include anesthesia and surgery accidents, post-operative hemorrhage and genital infections mainly, as well as HIV infections occurring within the 6 weeks wound healing requires after medicalized circumcision. Those infections will be detected by comparison of HIV serological results at M.0 and M.3, and analyzed with between-group comparison.

Randomization
All participants – meeting the inclusion criteria in absence of non-inclusion criteria - will be randomly assigned to medicalized circumcision. Randomization will be stratified by centre. The intervention group patients will be sent to a physician entitled for the medicalized circumcision designed in this trial.

Questionnaires
Questionnaires will be developed for M.0 (inclusion) and M.3 periods, and for M.12 and M.21 follow-ups. They will be built up from the questionnaires this project team has already used in South Africa: those are adapted from the questionnaire proposed by UNAIDS [37]. They will include general questions, queries about sexual behavior and condom use, as well as questions regarding possible STDs and their treatments. During the trial, questions about sexual behavior will include description of all sexual partners within the period (each partner characteristics, sex acts description and report of condom use for each partner).

Laboratory tests and clinical examination
Venous blood samples will be collected at M.0, M.3, M.12 and M.21. HIV tests will be applied to these samples (2 different ELISA assays with Western Blot when one test is negative) as well as HSV-2 tests (2 different ELISA including MRL assay). Detection of syphilis will be made at M.0 (RPR test and TPHA). 3 aliquot parts will be prepared from each

serum. The first one will be used for biological analyses, the second one could help creating a serum bank in the National Institute for Virology (NIV) of South Africa and the third one will be sent to Paris. All participants will undergo genital examination at M.0, M.3, M.12 and M.21 for detection of genital ulcerations. The current status of circumcision will be assessed during these exams. If clinical STDs occur, participants will be sent to their geographical area health centers. Genital ulcerations require immediate swab collection and PCR analysis with Multiplex PCR assay (Roche Molecular system) in order to find out the ulceration etiology among the 3 following ones: HSV-2, Treponema Pallidum (syphilis) and Hemophilus Ducreyi (chancroid). Clinical examinations and swab collections will be carried out by equally trained nurses. Within the trial period, beyond the M.12 and M.21 scheduled clinical exams, all participants will be asked to undergo treatment for any occurring STD, in their home STD clinics here an out-patient form will be filled by the health center staff; if any genital ulceration, swab collections will be carried out and analyzed like the ones performed at M.12 and M.21. Those health centers providers will follow a standard training in order to ensure the quality of recorded data.

Counseling
All candidates for participation and participants, will receive prevention counseling concerning HIV infection and other STDs at M.0, M.3, M.12 and M.21. Counseling will be received from specialized staff and will include general information about STDs and their transmission, their prevention (condom use, genital hygiene, reduction of partners number), and the importance of their treatments. All participants and candidates for participation will benefit of free condom delivery, and condoms will be freely available in each center. Lastly, counseling will make the possible role of male circumcision clear, it will inform participants that the maximum expected outcome is a partial protection only and absolutely not a total protective effect.

Follow-up of participants
Every participant who did not come to the follow-up study centre should receive two reminder letters. In case of failure, submission of questionnaires, collection of blood samples (on filter paper if necessary) and clinical exams will take place at home. People about to move should require particular attention in order to record their new addresses. In any event, people leaving the geographical study area, should be asked to keep participating in the trial on the condition that free transport will be provided for them.

Sequence of analysis

All participants will undergo an interview using a questionnaire, a clinical examination and a blood sample at M.0, M.3, M.12 and M.21.

- Analyses of the M.0 questionnaires and serologies will make it possible to compare the 2 groups concerning HIV, HSV-2, syphilis and sexual behavior (including condom use).
- Comparing HIV incidence between M.0 and M.3 will allow checking that this incidence does not increase in the intervention group in relation to medicalized circumcision and the healing period.
- Comparing HIV incidence between M.0 and M.12 will make it possible to perform intermediate analyses which might bring the study to an end if the differences of HIV incidence were statistically significant between the intervention group and the control one.
- Comparing HIV incidence between the intervention group and the control one within the M.0-M.21 period, will allow us to meet the main objective of this study.

- Comparing HSV-2 and genital ulcer disease incidence, as well as sexual activity (number of new partners, number of short-term partnerships, number of sex acts, condom use) between the intervention group and the control one within the M.0-M.21 period will allow us to meet the secondary objectives of this study.

Limitation of biases

Everything possible will be done to avoid biases. Thus, special attention shall be given in order to minimize the number of lost participants by sending reminder letters (see above description of the participants follow-up). Both groups' management will be identical concerning counseling, clinical exams and questionnaires submission. Except for the person in charge of clinical exams, people chosen for follow-up will be unaware of the participants groups. Moreover, as male circumcision will be performed without double blind, the IMC will be the only authority within the study to be informed of the outcome in both groups.

Confidentiality

Questionnaires as well as results of HIV and HSV-2 tests will be confidential and transmitted to the team in charge of statistical analyses only. Questionnaires and laboratory findings will be linked by means of a code. The results of the M.0 syphilis test will be disclosed to participants.

Statistical analysis

Statistical analysis and presentation of results will follow the classical recommendations for randomized studies [38] and will take account of recent recommendations in this field [39]. Thus, univariate comparison between the 2 groups will be a significant outcome, factors for adjusting are yet predetermined and no subgroups analyses will be performed but some interactions will be checked.

Men will be selected independently of their HIV serologic status. But the main objective is comparing HIV incidence in the 2 groups within the M.0-M.21 period, among the M.0 HIV seronegative men. An Intention-to-treat analysis will be performed. The effectiveness (in %) of medicalized circumcision will be characterized by 100x(1-RR), RR being the relative risk of HIV among circumcised men comparatively to uncircumcised men. In both groups, incidences are numerically equal to the seroconversion percentage among the M.0 HIV negative men in each group within the M.0-M.21 period. The comparison will be carried out using the bilateral Fisher exact test. The level of significance has been set to 5% and the intermediate analysis level of significance will be determined with the O' Brien method [40].

In a complementary analysis, the effect of medicalized circumcision on HIV infection will be adjusted for the M.0 and M.21 HSV-2 status, marital status, age, number of sexual partners, occurrence of genital ulceration within M0 and M21, condom use and finally the study centre. This analysis will be carried out using logistic regression.

No subgroup analysis will be performed but medicalized circumcision effects could vary according to the HSV-2 status; thus, interactions between circumcision and HSV-2 will be studied within the multivariate analysis of HIV status.

Moreover, a multilevel longitudinal analysis (visit, individual) will be carried out in order to take account of the repeated measurements. This sort of model may also give simultaneous explanations for several responses (HIV, HSV-2, genital ulcerations) [41].

Analysis of the effect of medicalized circumcision on HSV-2 and genital ulcerations will be just the same as for HIV effect.

A Fisher exact test or a Chi-squared test for qualitative variables, and nonparametric Mann-Whitney test for quantitative variables, will be used to check a possible HIV incidence increase between M.0 and M.3 in the intervention group by comparison to the control one. Moreover, multivariate analyses should be carried out with adjusting for age and marital status.

Population characteristics

The study will be undertaken in the general population of the townships of Johanneburg area around the cities of Sebokeng, Evaton and Orange farm. This population is expected to have quite similar socio-demographic characters to the population of Carletonville. In this last city, the HIV epidemic is well documented [33, 42]. In 1999 the prevalence of HIV and HSV-2 infections among men aged 18-24 years was respectively 15.4% (65/421) and 24.4% (102/408). At 18 years, this prevalence was respectively 3.3% (3/90) and 11.1% (10/90), and it reached 40.0% (20/50) and 50% (25/50) at 24 years. The incidence of HIV and HSV-2 infections between 18 and 24 years can be estimated to about 5.24% ((40.0-3.3)/7) and 48.4% ((50.0-11.1)/7). From a preliminary survey in Orange Farm, the HIV incidence among men can be estimited to 2.7%, a value lower than the value in Carletonville.

Number of subjects

Due to counseling and condom distribution in both study groups, the annual incidence of HIV infection in the control group will decrease by about 20%, thus reaching 2.7% X 0.80 = 2.2%. After the 21 month follow-up, the HIV prevalence among the M.0 HIV negative subjects of the control group will be thus 2.2% X 21/12 i.e. 3.9%. In the intervention group, the effect of medicalized circumcision should allow a 50% reduction compared with the control group. After 21 months, the expected HIV prevalence among the M.0 HIV negative subjects should be thus 2.0% in the intervention group. In order to detect a 3.9% to 2.0% variation when the level of significance is 5% and the power 80% using the unilateral Fisher exact test, the number of subjects has to be 1300 in each group.

If about 15% of the study subjects are to be lost in each group after 21 months, and to take account of the 13% HIV prevalence of the enrolled subjects, in order to obtain at the end of the 21 months 850 participants which were HIV negative at enrollment, it is thus necessary to recruit 1300/(1-0.15)/(1-0.13); i.e. approximately 1760 participants in each group. It will be thus necessary to recruit 3520 participants in all.

As a comparison, the Robert Bailey protocol, which is financed by the NIH, plans to recruit 2000 subjects for 24 months of participation.

Feasibility and mode of recruitment

The choice of a multi-centre study is necessary to ensure the feasibility of recruitment. Indeed, the population of the townships in the region 11 and around Sebokeng is estimated at about 600 000 people. There are about 36 000 men aged 18-24 years (6% of the whole population). The study thus requires to recruit about 14 % (3520/24500) of all men aged 18-24 years.

A population based survey led in Carletonville among a representative sample of 606 men (see above) let us estimate that about 9.5% of all men aged 15 to 24 years are circumcised with a median age of 17 years at circumcision. Moreover, about 59% of uncircumcised men declare to be willing circumcision if it could guarantee them some protection against STDs. Moreover, the medicalized circumcision which will be performed during this study meets the usual practices of the concerned areas, for the age at circumcision as well as for the place to carry it out.

Practical organization

In each of the 3 centers involved in the study, during the phase of recruitment of participants, a 2 persons team will be committed to inform the general population, to give the information leaflet to volunteers, to answer all their questions and collect their written consents. In each city a centre will be opened in order to meet and follow-up all participants. These centres will have a room for reception of participants, another one for the management of questionnaires and another one for clinical exams and blood tests. Each centre will be staffed with a secretary and a person in charge of the reminder letters. Recruitment, inclusion phase and follow-up of participants (questionnaires, clinical exams, counseling and blood tests) will be carried out by a 3 persons mobile team (investigator, nurse, counselor) who will share their time between the 3 centres. The numbers of recruited people will be similar in each centre (about 800-850).

Budget

Staff. The staff includes center employees (secretary, person in charge of the reminder letters), the mobile team members (driver, nurse, counselor and investigator) and the coordination centre members (full-time coordinator, full-time secretary, part-time laboratory technician). It will also include part-time monitoring and supervising staff: physician (10% time) and logistician (10% time). The staff is required for total duration of the study (30 months). During the 9 months of inclusion/recruitment phase, two additional persons in each center are required to inform the general population and candidates for participation.

Equipment. The only equipment necessary to this project is a vehicle to transport the mobile team and carry the material (biological samples and questionnaires).

Operating costs. Mainly, these include the laboratory tests costs, the circumcisions costs, the participants indemnities, the expenses related to the buildings (hiring, working) and to the use of the vehicle.

Ethical considerations (according to references [43-45]

Justification for the study

Objectives and design of the study are justified by our inability to predict the effect of medicalized circumcision in young men aged 18 to 24 years in South Africa, and by the potentially considerable interest in demonstrating a protective effect against HIV, HSV-2 or genital ulcerations. One cannot exclude a possible increase of HIV incidence in the intervention group due to vulnerability of patients to infection during the healing period and a possible change in sexual behavior within the intervention group related to the unfounded belief that medicalized circumcision is totally protective.

Including participants independently of their HIV and HSV-2 status

This study attempts to highlight a protective effect of medicalized circumcision on a group of men as similar as possible to those who will be eligible for this method of relative prevention in case of a successful study outcome. Thus the survey will analyze the effect of medicalized circumcision on HIV, independently of the initial HSV-2 status, as well as the effect of

medicalized circumcision on HSV-2, independently of the initial HIV status. However, complementary analyses will be performed, which will take account of the initial serologic status. Moreover, considering the insignificant success of the anonymous and free Screening Units, as well as the insignificant number of participants asking for the results of their HIV tests during cross-sectional surveys, it would be impossible from a practical point of view, to carry out this study if a negative HIV serology was required as an inclusion criterion. In addition, excluding of the study all positive HIV men or positive HSV-2 ones would not only lead to discrimination but above all to the risk of stigmatizing excluded men.

HIV tests

At inclusion, all participants will be informed of the nature of biological tests that will be performed during the study. At each blood sample collection participants will be given opportunity to know their HIV status. In this case, counseling services will take place before and after the test. All patients diagnosed with HIV through voluntary serology will be treated in local STD clinics using the protocols applied in South Africa.

Before study

Ethical approval should be obtained from the ethics Committee of the University of Witwatersrand. The protocol will be modified if necessary according to the Committee requests. This project will be submitted to the Medicine Control Council of South Africa, which is responsible in this country for clinical research authorizations. The project will be thoroughly discussed with the political leaders of involved cities and with concerned community leaders. Visiting a traditional doctor or attending an "initiation school" will be compatible with participation so long as circumcision will be performed by physicians according to this study protocol. Volunteers will be recruited by means of advertisement, leaflets, reunions and local meetings. Every eligible man should receive information leaflets concerning the study. Every potential participant will be given clear and full explanations of the constraints involved, laboratory tests to be performed and questionnaires to be answered. Before randomization, participants will have to sign a written consent form. Before giving their written consent, candidates will have a 5 days period to make up their mind.

During study

Participants will be able to withdraw at any time during study without any explanation. Any clinical abnormality or positive syphilis serology will result in sending the concerned participant to a Health centre where to receive conventional treatments [46]. The study team will have to check in each Health Centre before and within the trial, compliance with national recommendations and availability of necessary drugs. The study design includes the management of medicalized circumcisions, performed at the beginning of study for the intervention group, or at the end of study for the control group, and the handling of any occurring adverse effect of circumcision. Transport will be provided free of charge from home to the study centre for participants, who will receive an honorarium for each of the M.0, M.3, M.12 and M.21 visits, that should be equal to the average for one working-day in the area.

Submitting questionnaires and performing clinical examinations will require protections for privacy of persons and confidentiality of health information. Questionnaires and biological results will be connected by means of a code. However the link between one code and one participant will be confidentially kept by the person in charge of the study. Answers to questionnaires and biological results (HIV and HSV-2) will be disclosed to the statistician only who will sign an agreement of confidentiality. A prevention counseling will take place initially for every candidate to participation and during each visit of participants. It will

concern STDs in general and more particularly HIV infection. Any adverse effect appearing between M.0 and M.3 as well as the results of the M.12 intermediate analysis are to be communicated to the IMC who might possibly urge the Scientific Council to discontinue the study. The follow-up of subjects should require a very particular care in order to limit the number of lost participants. For this purpose, a specific person will take charge of reminder letters.

At the end of study

A scientific article will be written as soon as the study results are known. Main signatories will be Bertran Auvert, Adrian Puren, Emmanuel Lagarde, Dirk Taljaard, Philippe Aegerter, Mohamed Haffejee and Lynn Morris.

The study results will be notified to medical and political authorities and to community leaders of the cities in which the study was carried out. If the study shows a protective effect of medicalized male circumcision, this operation will be proposed to the control group participants. Moreover, if the study shows a protective effect of medicalized male circumcision, funds will be required in order to lead a campaign for medicalized male circumcision in the cities where the study was carried out.

Finally, the whole anonymized data (i.e. data lacking of the participants code) will be given to the National Institute for Communicable Diseases of Johannesburg (South Africa)

3 General management

Membership

Investigator-Research coordinator:
Prof Bertran Auvert (INSERM U88)
Investigators:
Dr. Adrian Puren (Biologist, National Institute for Communicable Diseases (NICD), South Africa)
Mr. Dirk Taljaard (Logistician, Progressus CC, South Africa)
C*entre of analysis and scientific management of the study*
INSERM U88
Methodologist - Biostatistician
Dr. Philippe Aegerter (INSERM U88, France)
Epidemiologist
Dr. Emmanuel Lagarde (INSERM U88, France)
Laboratory performing the study biological tests
NICD (South Africa)
Coordination of surgical procedures
Pr Mohamed Haffejee (Urology, University of Witwatersrand, South Africa)
Monitoring in South Africa
Progressus cc (South Africa)

Scientific Council *(subject to confirmation of participation)*
Prof. Bertran Auvert (INSERM U88, France)
Prof. Robert Bailey (USA)
Dr Daniel Halperin (USAID, USA)
Dr. André Bester (Goldfields Ltd, South Africa)
Dr. Michel Caraël (UNAIDS, Switzerland)
Dr. Benoît Ferry (IRD, France)
Prof. Abdool Karim (Medical Research Council, South Africa)
Dr. Emmanuel Lagarde (INSERM U88)
Pr Mohamed Haffejee (Urology, University of Witwatersrand, South Africa)
Dr. Lynn Morris (NICD, South Africa)
Dr. Adrian Puren (NICD, South Africa)
Prof. Roger Salamon (INSERM U, France)
Dr. Helen Weiss (London School of Hygiene and Tropical Medicine, the U.K.)
Prof. Brian Williams (WHO, Switzerland)
This committee will elect its president and will work by e-mail.

Independent Committee of Monitoring (ICM) *(subject to confirmation of participation)*
Clinician: Dr. Nicolas Dupin (Hospital Tarnier, Paris)
Biostatistician: Dr. Bernard Asselain (Institute Curie, Paris, France)
Ethician: Prof. Peter Cleaton-Jones, (Wits University, South Africa)

Diagram of the study

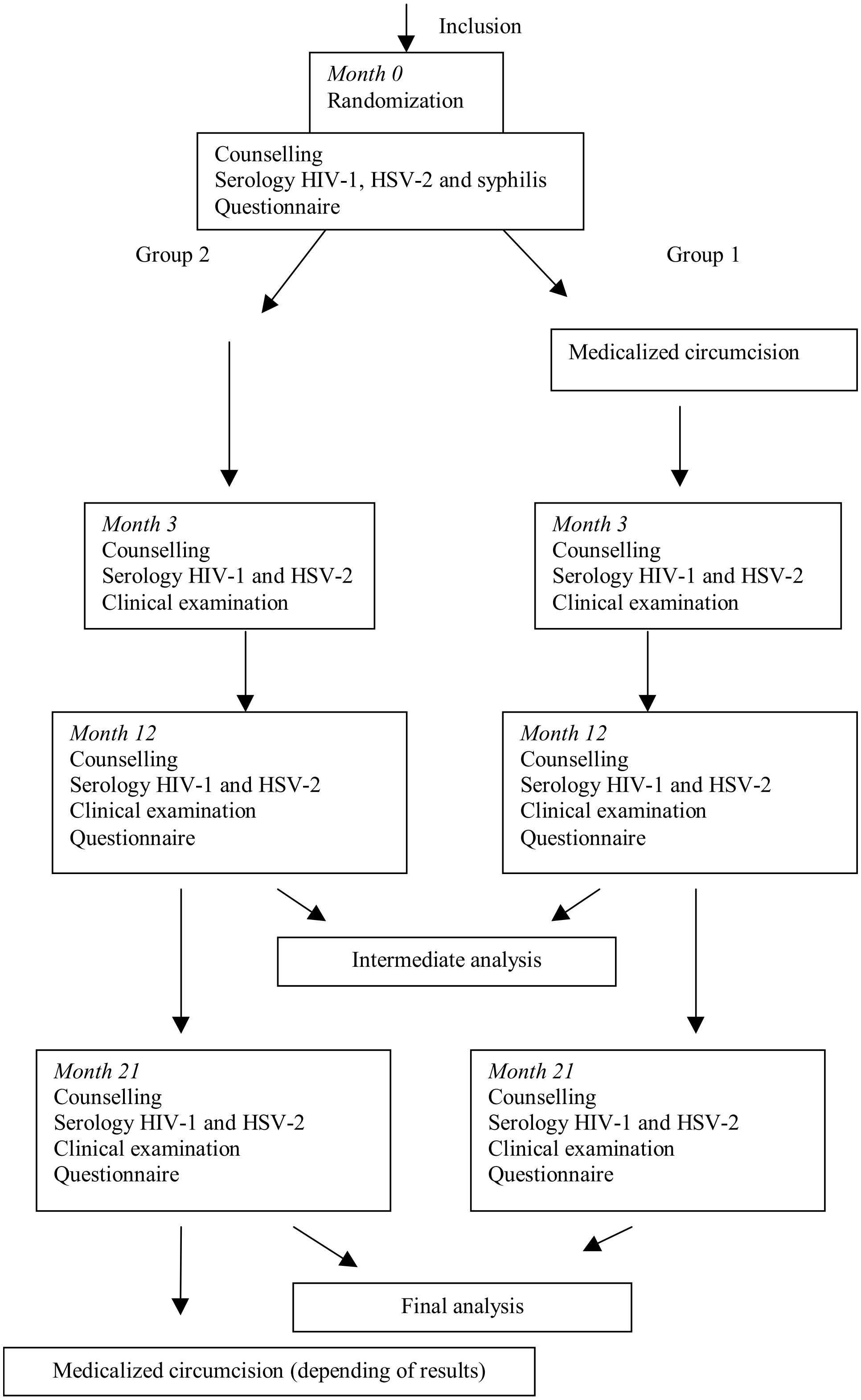

References.

1. Moses, S., J.E. Bradley, N.J. Nagelkerke, A.R. Ronald, J.O. Ndinya-Achola, and F.A. Plummer, *Geographical patterns of male circumcision practices in Africa: association with HIV seroprevalence.* Int J Epidemiol, 1990. **19**(3): p. 693-7.
2. Bongaarts, J., P. Reining, P. Way, and F. Conant, *The relationship between male circumcision and HIV infection in African populations.* Aids, 1989. **3**(6): p. 373-7.
3. Caldwell, J.C. and P. Caldwell, *The African AIDS epidemic [see comments].* Sci Am, 1996. **274**(3): p. 62-3, 66-8.
4. Cameron, D.W., J.N. Simonsen, L.J. D'Costa, A.R. Ronald, G.M. Maitha, M.N. Gakinya, M. Cheang, J.O. Ndinya-Achola, P. Piot, R.C. Brunham, *et al.*, *Female to male transmission of human immunodeficiency virus type 1: risk factors for seroconversion in men [see comments].* Lancet, 1989. **2**(8660): p. 403-7.
5. Diallo, M.O., A.N. Ackah, M.F. Lafontaine, R. Doorly, R. Roux, J.M. Kanga, P. Heroin, and K.M. De Cock, *HIV-1 and HIV-2 infections in men attending sexually transmitted disease clinics in Abidjan, Cote d'Ivoire.* Aids, 1992. **6**(6): p. 581-5.
6. Malamba, S.S., H.U. Wagner, G. Maude, M. Okongo, A.J. Nunn, J.F. Kengeya-Kayondo, and D.W. Mulder, *Risk factors for HIV-1 infection in adults in a rural Ugandan community: a case-control study.* Aids, 1994. **8**(2): p. 253-7.
7. Quigley, M., K. Munguti, H. Grosskurth, J. Todd, F. Mosha, K. Senkoro, J. Newell, P. Mayaud, G. ka-Gina, A. Klokke, *et al.*, *Sexual behaviour patterns and other risk factors for HIV infection in rural Tanzania: a case-control study.* Aids, 1997. **11**(2): p. 237-48.
8. Seed, J., S. Allen, T. Mertens, E. Hudes, A. Serufilira, M. Carael, E. Karita, P. Van de Perre, and F. Nsengumuremyi, *Male circumcision, sexually transmitted disease, and risk of HIV.* J Acquir Immune Defic Syndr Hum Retrovirol, 1995. **8**(1): p. 83-90.
9. Serwadda, D., M.J. Wawer, S.D. Musgrave, N.K. Sewankambo, J.E. Kaplan, and R.H. Gray, *HIV risk factors in three geographic strata of rural Rakai District, Uganda [see comments].* Aids, 1992. **6**(9): p. 983-9.
10. Simonsen, J.N., D.W. Cameron, M.N. Gakinya, J.O. Ndinya-Achola, L.J. D'Costa, P. Karasira, M. Cheang, A.R. Ronald, P. Piot, and F.A. Plummer, *Human immunodeficiency virus infection among men with sexually transmitted diseases. Experience from a center in Africa.* N Engl J Med, 1988. **319**(5): p. 274-8.
11. Auvert, B., Buvé A, Lagarde E., Chege J, Rutenberg N, Musonda R, Laourou M, Akam E, Weiss H, and f.t.S.G.o.t.H.o.H.E.i.A. Cities., *Male circumcision and HIV infection in four cities in sub-Saharan Africa.* Aids, 2001. **15**(suppl 4): p. S31-S40.
12. Gomo, E., P.P. Chibatamoto, S.K. Chandiwana, and C.T. Sabeta, *Risk factors for HIV infection in a rural cohort in Zimbabwe: a pilot study [see comments].* Cent Afr J Med, 1997. **43**(12): p. 350-4.
13. Lavreys, L., J.P. Rakwar, M.L. Thompson, D.J. Jackson, K. Mandaliya, B.H. Chohan, J.J. Bwayo, J.O. Ndinya-Achola, and J.K. Kreiss, *Effect of circumcision on incidence of human immunodeficiency virus type 1 and other sexually transmitted diseases: a prospective cohort study of trucking company employees in Kenya.* J Infect Dis, 1999. **180**(2): p. 330-6.
14. Quinn, T.C., M.J. Wawer, N. Sewankambo, D. Serwadda, C. Li, F. Wabwire-Mangen, M.O. Meehan, T. Lutalo, and R.H. Gray, *Viral load and heterosexual transmission of human immunodeficiency virus type 1. Rakai Project Study Group [see comments].* N Engl J Med, 2000. **342**(13): p. 921-9.
15. Rakwar, J., L. Lavreys, M.L. Thompson, D. Jackson, J. Bwayo, S. Hassanali, K. Mandaliya, J. Ndinya-Achola, and J. Kreiss, *Cofactors for the acquisition of HIV-1*

among heterosexual men: prospective cohort study of trucking company workers in Kenya. Aids, 1999. **13**(5): p. 607-14.
16. Urassa, M., J. Todd, J.T. Boerma, R. Hayes, and R. Isingo, *Male circumcision and susceptibility to HIV infection among men in Tanzania [corrected and republished in AIDS 1997 Mar;11(3):73-80].* Aids, 1997. **11**(1): p. 73-9.
17. Weiss, H.A., M.A. Quigley, and R.J. Hayes, *Male circumcision and risk of HIV infection in sub-Saharan Africa: a systematic review and meta-analysis.* Aids, 2000. **14**(15): p. 2361-70.
18. Auvert, B., Buvé A, Ferry B, Carael M, Chege J, Rutenberg N, Musonda R, Laourou M, Akam E, Weiss H, *et al.*, *Ecological and individual level analysis of risk factors for HIV infection in four urban populations in sub-Saharan Africa with different levels of HIV infection.* Aids, 2001. **15**(suppl 4): p. S5-S14.
19. Kelly, R., N. Kiwanuka, M.J. Wawer, D. Serwadda, N.K. Sewankambo, F. Wabwire-Mangen, C. Li, J.K. Konde-Lule, T. Lutalo, F. Makumbi, *et al.*, *Age of male circumcision and risk of prevalent HIV infection in rural Uganda.* Aids, 1999. **13**(3): p. 399-405.
20. Gray, R.H., N. Kiwanuka, T.C. Quinn, N.K. Sewankambo, D. Serwadda, F.W. Mangen, T. Lutalo, F. Nalugoda, R. Kelly, M. Meehan, *et al.*, *Male circumcision and HIV acquisition and transmission: cohort studies in Rakai, Uganda. Rakai Project Team.* Aids, 2000. **14**(15): p. 2371-81.
21. Cook, L.S., L.A. Koutsky, and K.K. Holmes, *Circumcision and sexually transmitted diseases.* Am J Public Health, 1994. **84**(2): p. 197-201.
22. Hart, G., *Factors influencing venereal infection in a war environment.* Br J Vener Dis, 1974. **50**(1): p. 68-72.
23. Parker, S.W., A.J. Stewart, M.N. Wren, M.M. Gollow, and J.A. Straton, *Circumcision and sexually transmissible disease.* Med J Aust, 1983. **2**(6): p. 288-90.
24. Jessamine, P.G., F.A. Plummer, J.O. Ndinya Achola, M.A. Wainberg, I. Wamola, L.J. D'Costa, D.W. Cameron, J.N. Simonsen, P. Plourde, and A.R. Ronald, *Human immunodeficiency virus, genital ulcers and the male foreskin: synergism in HIV-1 transmission.* Scand J Infect Dis Suppl, 1990. **69**: p. 181-6.
25. Wasserheit, J.N., *Epidemiological synergy. Interrelationships between human immunodeficiency virus infection and other sexually transmitted diseases.* Sex Transm Dis, 1992. **19**(2): p. 61-77.
26. Hayes, R.J., K.F. Schulz, and F.A. Plummer, *The cofactor effect of genital ulcers on the per-exposure risk of HIV transmission in sub-Saharan Africa.* J Trop Med Hyg, 1995. **98**(1): p. 1-8.
27. Fleming, D.T. and J.N. Wasserheit, *JOURNAL ???? From epidemiological synergy to public health policy and practice: the contribution of other sexually transmitted diseases to sexual transmission of HIV infection.* 1999.
28. Halperin, D.T. and R.C. Bailey, *Male circumcision and HIV infection: 10 years and counting [see comments].* Lancet, 1999. **354**(9192): p. 1813-5.
29. Szabo, R. and R.V. Short, *How does male circumcision protect against HIV infection?* Bmj, 2000. **320**(7249): p. 1592-4.
30. Lagarde, E., M. Carael, J.R. Glynn, L. Kanhonou, S.C. Abega, M. Kahindo, R. Musonda, B. Auvert, and A. Buve, *Educational level is associated with condom use within non-spousal partnerships in four cities of sub-Saharan Africa.* Aids, 2001. **15**(11): p. 1399-408.
31. Kamali, A., L.M. Carpenter, J.A. Whitworth, R. Pool, A. Ruberantwari, and A. Ojwiya, *Seven-year trends in HIV-1 infection rates, and changes in sexual behaviour, among adults in rural Uganda [In Process Citation].* Aids, 2000. **14**(4): p. 427-34.

32. Moses, S., F.A. Plummer, J.E. Bradley, J.O. Ndinya-Achola, N.J.D. Nagelkerke, and R. A.R., *Male circumcision and the AIDS epidemic in Africa.* Health Transistion Review, 1995. **5**: p. 100-3.
33. Auvert, B., R. Ballard, C. Campbell, M. Carael, M. Carton, G. Fehler, E. Gouws, C. MacPhail, D. Taljaard, J. Van Dam, *et al.*, *HIV infection among youth in a South African mining town is associated with herpes simplex virus-2 seropositivity and sexual behaviour.* Aids, 2001. **15**(7): p. 885-98.
34. *National HIV and syphilis sero-prevalence survey of women attending public antenatal clinics in South Africa 2000*, . 2001, Department of Health, Pretoria, South Africa. p. 1-21.
35. Taljaard, R., E. Lagarde, D. Taljaard, C. Campbell, C. MacPhail, B. Williams, and B. Auvert, *Potential for an intervention based on male circumcision in a town of South Africa with high levels of HIV infection.* Submitted for publication, .
36. Wynder, E. and S. Licklinder, *Tue question of circumcision.* Cancer, 1960. **13**: p. 442-5.
37. UNAIDS, *Looking deeper into the HIV epidemic: A questionnaire for tracing sexual networks*, in *Best practice collection, Key Material 98/27*. 1998, UNAIDS: Geneva. p. 1-24.
38. Begg, C., M. Cho, S. Eastwood, R. Horton, D. Moher, I. Olkin, R. Pitkin, D. Rennie, K.F. Schulz, D. Simel, *et al.*, *Improving the quality of reporting of randomized controlled trials. The CONSORT statement.* Jama, 1996. **276**(8): p. 637-9.
39. Assmann, S.F., S.J. Pocock, L.E. Enos, and L.E. Kasten, *Subgroup analysis and other (mis)uses of baseline data in clinical trials.* Lancet, 2000. **355**(9209): p. 1064-9.
40. O'Brien, P.C. and T.R. Fleming, *A multiple testing procedure for clinical trials.* Biometrics, 1979. **35**(3): p. 549-56.
41. Leyland, A. and H. Goldstein, *Multilevel modelling of health statistics*, ed. Wiley. 2001.
42. Gilgen, D., C. Campbell, B. Williams, D. Taljaard, and C. MacPhail, *The natural history of HIV/AIDS in South Africa: A biomedical and social survey in Carletonville*. Printed Matter, Braamfontein (South Africa) ed. 2000: Council for Scientific and Industrial Reasearch.
43. *International Ethical Guidelines for Biomedical Research Involving Human Subjects. Geneva, Switzerland.*, . 1993, CIOMS.
44. *UNAIDS policy on HIV testing and counselling. Geneva*, . 1997, UNAIDS, Geneva, Switzerland.
45. *Helsinki declaration. World Medical Association Declaration of Helsinki: Recommendations Guiding Medical Doctors in Biomedical Research Involving Human Subjects. Revised version.*, . 1989: Hong Kong.
46. *STD syndromic management*, . 1999, STD Reference Center, University of the Witwatersrand, Johannesburg, South Africa. p. 1-15.
