# Supplementary material for: Randomized, Controlled Intervention Trial of Male Circumcision for Reduction of HIV Infection Risk: The ANRS 1265 Trial
Source: PLoS Med. 2005 Oct 25;2(11):e298. doi: 10.1371/journal.pmed.0020298 (PMC1262556; doi:10.1371/journal.pmed.0020298)
Supplement: Supplementary file 2 [file pmed.0020298.st002.pdf]

Version2

Evaluation of the impact of voluntary, safe male circumcision on sexually transmitted infections - A randomised controlled trial (MC study)

Consent form

The original consent form stapled to the inclusion form
One copy to be given to the participant by the facilitator
Write the Participant Number on the participant copy:

Participant number : |__|__|__|__|__|__|__|__|__|__|__|__|

I have been informed by the person whose signature is given below, about the nature of the study 'Evaluation of the impact of voluntary, safe male circumcision on sexually transmitted infections', as well as what effects and possible advantages and possible adverse events or risk to expect. I received a copy of the written participant information and I had sufficient opportunity to ask questions and do not have any further questions at the moment.

My participation in this study is voluntary. I may end my participation in the study at any time without suffering any disadvantages. I am not required to give reasons for my decision. During my participation I will accept and follow the instructions of the centre staff and GP. I confirm that the information I have given on my health is correct.

I have been informed of the surgery that could be performed. I have been informed of the questionnaires about my life and my sexual behaviour that I will have to respond to. I have been informed of the blood samples that will be taken, of the medical examination I will have and the laboratory test that will be performed confidentially and anonymously on biological samples. I have been informed that a trained counsellor will be available during the duration of the study should I need to speak to a counsellor.

I have been informed and agreed that the participant records may be handled, stored and transmitted electronically and that data transferred to the study centre are entered in an anonymous way.

I agree to participate in the above-mentioned study.

Participant's name (in block letters)	Age	Date	Participant's signature

Interviewer's name (in block letters)	Date	Interviewer's signature (The person who conducted the consent discussion)

Version2

Evaluation of the impact of voluntary, safe male circumcision on sexually transmitted infections - A randomised controlled trial (MC study)

Consent form

The original consent form stapled to the inclusion form
One copy to be given to the participant by the facilitator
Write the Participant Number on the participant copy:

Participant number : |__|__|__|__|__|__|__|__|__|__|__|__|

I have been informed by the person whose signature is given below, about the nature of the study 'Evaluation of the impact of voluntary, safe male circumcision on sexually transmitted infections', as well as what effects and possible advantages and possible adverse events or risk to expect. I received a copy of the written participant information and I had sufficient opportunity to ask questions and do not have any further questions at the moment.

My participation in this study is voluntary. I may end my participation in the study at any time without suffering any disadvantages. I am not required to give reasons for my decision. During my participation I will accept and follow the instructions of the centre staff and GP. I confirm that the information I have given on my health is correct.

I have been informed of the surgery that could be performed. I have been informed of the questionnaires about my life and my sexual behaviour that I will have to respond to. I have been informed of the blood samples that will be taken, of the medical examination I will have and the laboratory test that will be performed confidentially and anonymously on biological samples. I have been informed that a trained counsellor will be available during the duration of the study should I need to speak to a counsellor.

I have been informed and agreed that the participant records may be handled, stored and transmitted electronically and that data transferred to the study centre is entered in an anonymous way.

I agree to participate in the above-mentioned study.

Participant's name (in block letters)	Age	Date	Participant's signature

Interviewer's name (in block letters)	Date	Interviewer's signature (The person who conducted the consent discussion)
