# Supplementary material for: Randomized, Controlled Intervention Trial of Male Circumcision for Reduction of HIV Infection Risk: The ANRS 1265 Trial
Source: PLoS Med. 2005 Oct 25;2(11):e298. doi: 10.1371/journal.pmed.0020298 (PMC1262556; doi:10.1371/journal.pmed.0020298)
Supplement: Supplementary file 3 [file pmed.0020298.st003.pdf]

Version 4

Evaluation of the impact of voluntary, safe male circumcision on STD infections in sub-Saharan Africa

A randomised controlled trial

MC study 01-07-2002

Participant Information sheet

Dear Sir,

You have been asked to take part in a clinical research study to assess the effect of a male circumcision. Male circumcision could reduce infection by STD's, but this has not been proven. It is therefore important to know that circumcision does NOT protect you from STD's. For this study we are looking for 3500 young men aged from 18 to 24 years.

Before you decide on your participation, it is important that you understand why this study is performed and what your participation in it involves. Please read the following instructions carefully, and if you have any question, don't hesitate to ask.

Background and purpose of the study

One of the possible reasons why the STD epidemic and HIV infections are high in some countries in Africa is the lack of male circumcision. Circumcision could partly prevent the infection by STD's. But it is not known if it does.

The main objective of this study is to evaluate if voluntary and safe male circumcision has a protective effect against infection with STD's and HIV.

Study surgery

The male circumcision will be performed in this study by trained general practitioners. They will follow the recommendations of the Department of Urology of the University of Witwatersrand. It will be done under local anaesthesia. It will be paid by the project.

Attribution of medication

In order to evaluate the effect of safe male circumcision, we will have to ask some of you to be circumcised at the beginning of the study, and others at the end of the study. The allocation of participants in the 2 groups will be done by chance.

Expected adverse events related to the study medication.

The circumcision surgery is safe. But it will not be possible to be circumcised if you are sick or if you have any disease that is incompatible with surgery.

Disadvantages of the participation in the study

An intravenous blood sample (1 tube of 10 cc) will be taken 4 times. This may cause a little bleeding and some pain for a few seconds.

When you are circumcised you will be asked to have no sexual contact in the 6 weeks after surgery. To have sexual contact before your skin of your penis is completely healed, could lead to infection

if your partner is infected with a sexually transmitted disease. It could also be painful and lead to bleeding. If you desire to have sexual contact in the 6 weeks after surgery, despite our recommendation, it is absolutely essential that your use a condom.

You could have some pain after the surgery. In very rare cases, the surgery could lead to infection, allergic reaction to the anaesthesia. In extremely rare cases mutilation and death could occur, but this is not expected in this study because trained medical doctors who have been selected by the study team perform the surgery. Care will be taken to prevent and treat such side effects if they occur.

Advantages of the study medication and participation in the study

If this study shows that voluntary and safe male circumcision reduce transmission STD's or HIV, you will have contributed to important progress in the fight against STD's and HIV.

During the study you will have some medical examinations and biological tests that could detect diseases that will be treated free of charge.

Trained counsellors will be available for the duration of the study to discuss any problems or provide emotional support to you should it be required.

Course of the study

Before any study procedures can be performed, you will be asked to sign a consent form.

The duration of this study is 21 months and will include 4 visits.

At the first (initial) visit
Your general health is checked.
A blood sample (10cc) will be taken
You will have to answer a questionnaire.
You will know if you will be circumcised at the beginning or at the end of the study.

Safe male circumcision will then be performed for half of the participants (selected by chance).

At the second visit (3 months after the beginning)
You will have a medical examination
A blood sample (10cc) is taken

At the third visit (12 months after the beginning)
You will have a medical examination
A blood sample (10cc) is taken
You will have to answer a questionnaire

At the fourth visit (21 months after the beginning)
You will have a medical examination
A blood sample (10cc) is taken
You will have to answer a questionnaire
Safe male circumcision is then proposed for the participants who have not been circumcised at the beginning of the study.

At each visit you will receive information on sexually transmitted diseases and how to avoid these diseases. A swab will be used in case of a genital ulceration.

If you have not been circumcised at the beginning of the study, you will normally be circumcised at the end of the study. In case the study do not show any protective effect of male circumcision on sexually transmitted, you will be informed. It will not be useful for you to be circumcised in this case. However should you still prefer to be circumcised you will then be circumcised.

Biological tests

A detection of infection by syphilis will be performed each time a blood sample is taken (at visits 1, 2, 3 and 4). If you are infected the result will be communicated to you and you will be treated. If a genital lesion due to syphilis or other treatable disease is detected at visits 1, 3 or 4, you will be informed and it will be treated.

HIV and herpes detection will be done each time a blood sample is taken. The results will be kept absolutely confidential and will not be linked to your name. It will be communicated only to the unit in charge of the analysis of the study located outside of South Africa.

In case of genital lesion, the swab will be analysed for HSV-2, syphilis and chancroid.

Responsibility of the participants

After you have decided to participate in this study, you are requested to be present at each visit. If you are moving out in the same town or in another town you are requested to indicate to the MCT Centre your name, your old and new addresses. To facilitate the course of this study you are requested to have with you at each visit the card that will be given to you at the first visit. It is important for the study that you continue to participate even if you move out.

If you are allocated in the group of people who will be circumcised at the beginning at the study, it is essential that you follow our instructions and that you go for circumcision to the Doctor that we will indicate to you in the days following your inclusion in this study.

The participation in this study is not expected to cause any restrictions to your daily living.

Participation in the study, withdrawal from the study and benefits

Your participation is completely voluntary, and if you decide to participate, you may withdraw from the study at any time without giving a reason and without incurring displeasure or penalty

Confidentiality

In the data stored in an electronic database and transferred to the persons in charge of the analysis of this study, you will be identified with a number. Your personal data (name and address) are retained at the study centre. Your personal data can be reviewed by local and foreign health authorities, as well as by an independent ethics committee. This is to compare original data with those analysed to ensure proper documentation of the study.

Compensation

For your kind participation you will receive some money. You will receive 30, 40, 80 and 150 Rand at the visit 1, 2, 3 and 4,

respectively. You will therefore receive a total of R 300 for your participation.

In case of injury due to your participation compensation will be offered. This compensation will be proposed by the persons in charge of this study and could be discussed with the ethical committee.

Long term evaluation

After the end of this study you could be asked to participate in a future evaluation. This following study will evaluate the long-term impact of the safe male circumcision that you have received during this study.

Schedule and supervision

Before the study is started, this Participant Information Form and Consent Form, including the study plan, have been approved by the Ethics Committee of the South African Medical Research Council (on the 22 February 2002) and by South African medicines Control Council. These Ethic Committee and Authorities will also be informed of all possible changes in the study plan.

According to the study plan, the study will be started in 2002, and will be conducted in 3 towns of South Africa.

For further information and/or if you have any problems, please leave a message for us at the MCT Centre or the Medical Doctor who performed your circumcision.

You could also contact the following physician (directly or by leaving a message at the study centre):
Name: Doctor Adrian Puren
Tel:(a) (011) 321 4228, (b) 082 908 8048
Reverse charges call can be made to anyone of these numbers

The MCT Centre can be contacted at: 082 415 6945

You could also contact the Ethical committee of Wits University:
Name: Prof. Cleaton-Jones
Tel: (011) 717 1234

In case of emergency, please call the physician who is looking after you during this study. His name and telephone number will be given to you at the beginning of the study.

If you decide to participate in this study, please keep this Participant information form.

Thank you.
